# Applicability and Suitability of the Embryological–Clinical Classification of Female Genital Malformations: A Systematic Review

**DOI:** 10.3390/jcm13102988

**Published:** 2024-05-19

**Authors:** Victoria Navarro, Maribel Acién, Pedro Acién

**Affiliations:** 1 Obstetrics and Gynecology Service, Elda University Hospital, 03600 Elda, Alicante, Spain; victoria.nl@coma.es; 2 Reproductive Biopathologies Mixed Research Unit FISABIO-UA-UMH, 03550 San Juan, Alicante, Spain; 3 Obstetrics and Gynecology Service, San Juan University Hospital, 03550 San Juan, Alicante, Spain; 4 Division of Gynecology, Miguel Hernández University, Campus of San Juan, 03550 San Juan, Alicante, Spain; acien@umh.es; 5 Grant Next Generation EU-EGA Institute for Women’s Health, University College London, London WC1E 6DE, UK

**Keywords:** genital malformations, müllerian, embryological–clinical classification, female genital tract, renal agenesis

## Abstract

Complex urogenital malformations are clinically highly relevant; thus, they must be appropriately diagnosed and classified before initiating treatment. **Background/Objectives:** This study aimed to evaluate the applicability and suitability of the embryological–clinical classification of female genital malformations. **Methods:** A systematic review of cases of genital malformations reported in the literature from 2000 to 2020 was conducted. Case reports and series with the following combinations: “female genital tract” AND (malformation OR anomaly OR müllerian anomaly OR uterine anomaly OR cervical anomaly OR vaginal anomaly OR cloacal anomaly OR urogenital sinus); and “female genital tract” AND (renal agenesis OR ectopic ureter) were searched. A total of 3124 articles were identified, of which 824 cases of genital malformation were extracted. The characteristics of each malformation were included in a database for further analyses. **Results**: Using the embryological–clinical classification, 89.9% of the published cases and 86.5% of the 52 cases defined as unclassifiable by their authors have been classified in this review. In 73 cases (72.2%), the classification of the malformation using the AFS system was incomplete because although the type of uterine anomaly of the AFS classification matched that of the embryological–clinical classification, characteristics of the urinary system or the vagina were overlooked when using the AFS system. Following a dispersion matrix, we have been able to show that the embryological–clinical classification system is able to classify and subclassify the genitourinary malformations more accurately. **Conclusions:** The applicability of the embryological–clinical classification has been confirmed after classifying most of the cases of genital malformation previously published. This system also provides a more complete and accurate classification than other classifying systems exclusively based on Müllerian duct development or uterovaginal parameters, demonstrating its suitability.

## 1. Introduction

Female genital malformations commonly occur in 3% of all women, 4% of infertile women, and 15% of those who recurrently experience miscarriages [1]. However, and although they are not always detected, these frequencies mainly refer to common genital malformations that affect the uterine cavity and have an impact on reproductive outcomes.

On the contrary, complex genital malformations which also affect the urinary system or other levels of the genital tract are rare but may lead to significant gynecological symptoms that severely affect the patient’s quality of life, specially at adolescence when menses start. Classifying female genital malformations requires knowledge of embryology of the female genital tract, of the involvement of all elements derived from the urogenital ridge, and of the Müllerian duct development, fusion, and resorption processes. The scientific literature tends to follow classifications based on Müllerian duct development, such as the American Fertility Society (AFS) system [2], recently updated in the American Society of Reproductive Medicine (ASRM) classification [3], or the European Society of Human Reproduction and Embryology (ESHRE)/European Society for Gynaecological Endoscopy (ESGE) [4] consensus on the classification of female genital tract congenital anomalies. However, these classifications are solely Müllerian (utero or uterovaginal) and provide no information regarding the origin of the malformations. Conversely, the embryological–clinical classification of female genital malformations proposed by Acién in 1992—modified in 2004 [5] and updated in 2011 [6]—is based on the correlation between the embryological origin of the malformation and its anatomical anomaly observed throughout the female genital tract, including the urinary system, for a complete diagnosis, with the goal of proposing the most appropriate typification and adequate therapeutic intervention (full classification available in Supplemental Material).

Several embryological parameters should be considered to adequately manage female genital malformations. The appropriate development, fusion, and resorption of the separating wall between both Müllerian ducts seem to be induced by the Wolffian ducts. The fused Müllerian ducts form the uterus up to the external cervical os, and the inducing mesonephric ducts form the sinuvaginal bulbs, incorporate the Müllerian tubercle’s cells, and give rise to the vaginal plate [7,8]. Because the ureteral bud sprouts from the opening of the Wolffian duct in the urogenital sinus, the absence or distal injury of one of these ducts will give rise to renal agenesis, a blind or atretic ipsilateral hemivagina and a uterine anomaly (fusion or resorption defect), due to a failure of the inducing function of the injured Wolffian duct. In the absence of the formation and caudal growth of the urogenital wedge, there is persistent urogenital sinus, and then, the opening of the vagina into the sinus can be seen as a vesicovaginal fistula just underneath and between both ureteral orifices [6]. Interruptions in this embryological process at any point may lead to various congenital anomalies of the reproductive system [9]. Because the formation of the urinary system is closely related to the formation of the reproductive tract, abnormalities of the kidneys, ureters, or bladder are often associated with abnormalities of the genital tract. In a study, Diehl et al. [10] stated that further evaluating the urinary system is important secondary research. However, this evaluation is not secondary research but a fundamental part of the research of malformations, given the relationship between the genital and urinary systems, which share a common embryological origin.

This study aimed to assess the advisability of the embryological–clinical classification of female genital malformations [6]; for this purpose, the applicability and suitability were determined. Therefore, we conducted a systematic review of cases of genital malformation reported in the literature for 20 years to determine whether these cases had been classified or not using the AFS [2] or ESHRE/ESGE [4] systems and tried to classify them with the embryological–clinical classification. We also checked if this system was more specific when typifying the anomalies.

The objectives of this study were to determine the following:

- applicability of the embryological–clinical classification [6] by assessing the percentage of malformations that can be classified using this method following the details of the anomaly as described in the articles retrieved from the literature.
- suitability of the embryological–clinical classification [6] by assessing whether it is more complete and more accurate when applied to a malformation with respect to the AFS [2] or ESHRE/ESGE [4] classification systems.

## 2. Materials and Methods

A systematic literature research was performed in the PubMed and Cochrane Databases, including 20 years (from 2000 to 2020) and all case reports and case series with the following combinations:

- “female genital tract” AND (malformation OR anomaly OR müllerian anomaly OR uterine anomaly OR cervical anomaly OR vaginal anomaly OR cloacal anomaly OR urogenital sinus).
- “female genital tract” AND (renal agenesis OR ectopic ureter).

This systematic review commenced in February 2021 and was completed in April 2023. The selection process started with a total of 3124 articles. This process included two exclusion phases: the first phase consisted of selecting articles by title that showed the presentation of a malformation, which was independently performed by the three authors, and collecting the articles that had been selected by at least two of the three authors, thus narrowing the search to 1219 articles (39% of the initial sample); the second phase consisted of selecting articles, by summary, whose content focused on the description of the malformation, limiting the search to 858 research articles (27% of the initial sample), which have been individually evaluated for data extraction. This second phase was carried out by one author, and the others checked for agreement.

**Figure 1:**
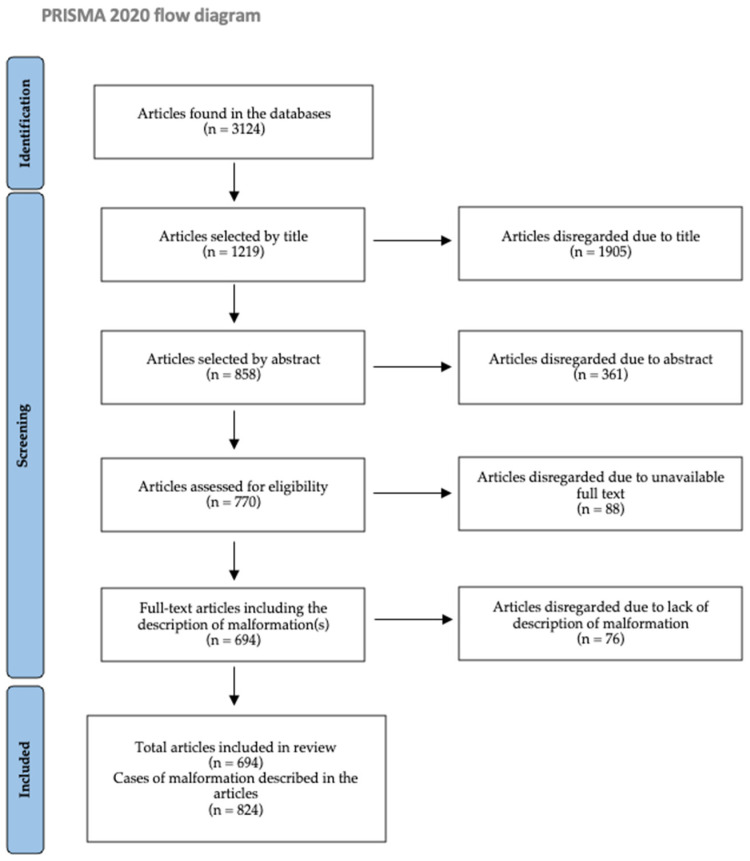
PRISMA flow diagram of the selection of articles and cases of malformation(s).

Of the 858 selected articles, the full text of 88 was inaccessible and, therefore, were disregarded for unavailability. The full text of the remaining 770 articles (24% of the initial total) was read, and 76 whose content did not include the description of the malformation were disregarded. Of the remaining 694 articles, we were able to extract a total of 824 described cases as some studies described various cases and types of malformations (Figure 1).

Each of these malformations was analyzed, and their descriptions, according to the following variables, were entered into an Excel spreadsheet (Excel Version 16.85 (24051214), Microsoft Office for Mac, Madrid, Spain):

- Characteristics of the uterus
- Characteristics of the cervix
- Characteristics of the vagina
- Renal agenesis (RA)
- Diagnostic tests
- Classification (or not) of the malformation by the authors of the article and the system used for this purpose
- Classification of the malformation according to the embryological–clinical classification [6]
- Match (or not) between both classifications (the classification used by the authors of the article selected in the systematic review and the embryological–clinical classification)

The three authors commented on the most complex cases to reach a consensus on the classification of all malformations.

## 3. Results

### 3.1. Applicability

Of a total of 824 cases, the malformations had been classified by the authors of the articles in 159 cases (19.3%): using the AFS classification in 101 (63.5%) cases; using the embryological–clinical classification in 20 (12.6%) cases; using the ESHRE/ESGE classification in 16 (10.1%) cases; and using more than one classification in 22 (13.8%) cases. The authors had specified that they were unable to classify 52 malformations (6.3%): 32 (61.5%) using the AFS; 1 (1.9%) using the ESHRE/ESGE classification; 5 (9.6%) using any current classification; and 4 (7.7%) using an unknown classification system (not specified). Of the total number of cases, no classification was proposed in 613 malformations (74.4%) (Table 1).

After reviewing the descriptions of 824 cases of malformations, we classified 741 cases (89.9%) following the embryological–clinical classification and 559 (91.2%) of the 613 that were not classified by the authors in their articles (Table 2).

In total, 63 cases (7.6%) were grouped as “inconclusive” due to the lack of essential data in the descriptions of the malformation retrieved from the publication. This was a series of cases missing some key anatomical data, preventing the differentiation between two classification groups, primarily due to a lack of information on the urinary system. This lack of information precludes the differentiation between common Müllerian anomalies (group 3.A.2 of the embryological–clinical classification, which does not involve renal agenesis) versus mesonephric or Wolffian anomalies (uterine duplication with vaginal atresia or blind hemivagina and ipsilateral renal agenesis -group 2-, or unilateral genitourinary agenesis or hypoplasia -group 1.2-). In this group, a few cases lacked descriptions of the uterus, cervix, and/or vagina; thus, they might have involved more than one type of malformation, precluding the correct classification of the case [11,12,13,14,15,16,17,18,19,20,21,22,23,24,25,26,27,28,29,30,31,32,33,34,35,36,37,38,39,40,41,42,43,44,45,46,47,48,49,50,51,52,53,54,55,56,57,58,59,60,61,62,63,64,65,66,67,68,69,70,71]. In addition, 20 cases were not classified using the embryological–clinical classification system—four because we considered that a genital malformation was not demonstrated [72,73,74,75] and 16 because the case description lacked sufficient details to guide any classification.

Of the 52 cases defined in their respective papers by the authors as “unclassifiable”, we were able to classify 45 cases (86.5%) using the embryological–clinical classification. As for the remaining cases, after their revision, we concluded that one case was not a genital malformation and that the other six were incompletely described, preventing their correct classification. Table 3 shows the characteristics of the “unclassifiable” malformations according to their authors [10,76,77,78,79,80,81,82,83,84,85,86,87,88,89,90,91,92,93,94,95,96,97,98,99,100,101,102,103,104,105,106] and their classification group according to the embryological–clinical classification system.

Finally, uterine duplicity with a blind hemivagina (or atresia) and ipsilateral RA (malformation 2.1 of the embryological–clinical classification) were the most frequently described malformations in the literature, which accounted for 128 of the 731 classified cases, followed by 100 cases of urogenital sinus abnormalities.

### 3.2. Suitability

In this section, we analyzed the completeness and accuracy of the classifications provided in the original publications, assessing whether the original classification matched that of the embryological–clinical classification system. With the AFS classification, 101 cases of genital malformation were described (see Table 1). Among them, only 20 cases matched the embryological–clinical classification, eight did not match each other; in 73 cases [17,19,21,24,29,36,42,44,45,50,58,61,62,68,107,108,109,110,111,112,113,114,115,116,117,118,119,120,121,122,123,124,125,126,127,128,129,130,131,132,133,134,135,136,137,138,139,140,141,142,143,144,145,146,147,148,149,150,151,152,153,154,155,156,157,158], the classification of the malformation using this system was incomplete because although the type of uterine anomaly of the AFS classification matched that of the embryological–clinical classification, characteristics of the urinary system or the vagina were overlooked when using the AFS system. Thus, the embryological–clinical classification was more complete.

As for the ESHRE/ESGE classification, of 16 cases defined using this method (see Table 1), six matched the embryological–clinical classification, six did not match either classification, and four cases [159,160,161,162] presented an incomplete classification using the ESHRE/ESGE system by overlooking RA or failing to describe the cervix and the vagina.

Despite the clear trend of non-classification, AFS is the most frequently used classification system in the literature. However, the subtypes in the classification of female genital tract malformations that we were able to identify using the embryological–clinical classification are more specific or precise that those reported by authors using AFS. The dispersion matrix (Table 4) shows that we were able to more accurately classify and subclassify. For example, the 30 cases that were classified as type III according to the AFS system (didelphys uteri) could be subclassified according to whether these double uteri were associated or not with RA (1 isolated didelphys uterus), communication between both hemiuteri (1 not communicating), and characteristics of the vagina (23 blind), among other characteristics. In other words, the embryological–clinical classification provided a more accurate classification of female genital tract malformations than the other classification systems.

## 4. Discussion

This study shows a non-classification trend in the literature. Although AFS [2] has been the most used classification system, probably influenced by its publication year and the study period of this review, many other classification systems have been proposed for classifying complex female genital malformations (ESHRE/ESGE [4] and VCUAM [163], among others). However, only the embryological–clinical classification system analyses embryological alterations to understand malformations.

In 2015, Di Spiezio Sardo et al. [164] published a systematic review of cases of malformation. They claimed that the ESHRE/ESGE classification provided a complete description and classification of almost all known anomalies that had not been correctly classified using the AFS system. The authors grouped the cases retrieved from articles by type of malformation, not by number, which might have contributed to the favorable results. As they defined, a key characteristic of an “ideal” classification system is to be comprehensive, encompassing all possible variants and providing a clear description and classification. However, neither the ESHRE/ESGE nor the AFS classification systems can be considered comprehensive because they either classify anomalies overlooking embryological parameters, that is, without establishing a relationship between all components of the genitourinary system, and they are incomplete by being solely based on uterine or uterovaginal anomalies.

Towards solving this problem, the ASRM has recently published an update of the AFS classification aimed at maintaining the simplicity of the system while simultaneously expanding the classification to include anomaly categories [3]. Yet, surprisingly, this update has disregarded state-of-the-art genitourinary embryology concepts, instead focusing on morphological abnormalities of the Müllerian ducts and overlooking the entire urogenital crest, urogenital sinus, and gubernaculum [7,165]. Imaging the malformation should help us in the etiological and pathogenic diagnosis of the observed anomaly and possible associated anomalies. However, neither the recent American nor the European classification systems perform it. And images need to be appropriately interpreted, which cannot always be done without the correct context.

In this systematic review, instead, using the descriptions retrieved from articles published in the literature and the embryological–clinical classification, we have been able to classify a high percentage of anomalies (89.9%), even those previously defined as unclassifiable by their authors. Only the malformations with missing data on their description or a fundamental characteristic of their origin have not been classified in this study. These results demonstrate the high applicability and its diagnostic capacity by shedding light on aspects of the malformation that remain unclear when using other classification systems.

In addition, we also highlight the completeness and higher accuracy of the embryological–clinical classification. Using this system, we were able to differentiate one AFS [2] classification group into five groups. Therefore, according to data on the renal system, characteristics of the vagina or cervix, considered in the embryological–clinical classification, enable much more refining of the subclassification type within a group of uterine anomalies. This refinement may have implications for reproductive results, as demonstrated in the study by Acién et al. [166], which showed that the reproductive prognosis is more successful for a given type of uterine anomaly in patients with associated RA.

Although the usefulness of the embryological–clinical classification system is not the aim of this research as it has been previously addressed in other papers by our group [167], as shown in this study, the lack of data on the renal system prevents us from being able to adequately classify some malformations when using the embryological–clinical system. This and the anatomical details of the cervix and vagina are necessary for its use, to understand the reason for the anomalies, the correct interpretation of the images [168] or maybe even to find the most appropriate surgical solution for the patient [167] specially in the most complex cases [169,170,171].

The most complex cases are the most published in the literature. First, uterine duplicity with a blind hemivagina (or atresia) and ipsilateral RA, which account for 128 of the 731 cases classified in the literature, followed by urogenital sinus abnormalities (100 cases). In both situations, the authors most likely decide to publish a case report because of their striking description.

We have detected two published cases of genital malformations whose diagnosis has generated disagreement. Briosa et al. [172] have described a case of suspicion of Mayer–Rokitansky–Küster–Hauser (MRKH) syndrome for uterine duplication with a normal left hemiuterus and a right non-communicating cavitated rudimentary horn, single cervix and vagina, and right RA, suggesting not only a Müllerian but also a Wolffian anomaly (type 2.5 of the embryological–clinical classification [6]). Capito et al. [173] have described menstrual retention in a Robert’s uterus; however, the anomaly was a non-communicating cavity, with hematometra, and without connection to the fallopian tube, suggesting an accessory and cavitated uterine mass (type 4 of the embryological–clinical classification [6]), not an asymmetric uterine septum. Accordingly, we insist that knowing the correct genitourinary embryology is essential for studying, diagnosing, and subsequently treating genital malformations, especially complex malformations that lead to early gynecological and later reproductive problems, particularly in young adolescent patients [167]. This knowledge is maintained by the clinical–embryological classification system through the comprehension of the malformation as a whole.

### 4.1. Strengths and Limitations

Several case reports fail to describe the complete anomaly, ignoring crucial characteristics for its classification. Besides that, the most complex cases are the most published in the literature, indicating a bias.

On the other hand, the non-classification trend implies another limitation for this study, since this fact has prevented us from further study of the suitability assessment.

Some cases, especially those with anomalies that affect the connection between Müllerian ducts and Müllerian tubercle, may apparently not fit into the embryological–clinical classification, showing that this classification system necessitates additional review and update.

### 4.2. Interpretation

Female genital malformations may lead to significant gynecological symptoms. The malformation itself, as well as the management to solve the symptoms, can also compromise the reproductive health of patients. The embryological–clinical classification system supports the embryological origin of female genital malformations to better understand the clinical cases allowing an appropriate and individualized therapeutic approach [167].

Considering the above, the embryological–clinical classification system for female genital malformations may surpass the limitations of other classification systems because embryological analysis should be the basis for understanding and investigating malformations of the female genitourinary tract, especially complex anomalies. Knowing the embryological background may enable the physicians to more adequately counsel and treat each case [167].

This study demonstrates the applicability and suitability of the embryological–clinical classification system when classifying anomalies of the female genital tract. A system based on this foundation empowers physicians with an effective and comprehensive tool for classifying nearly all currently known anomalies of the female genital tract.

## 5. Conclusions

AFS has been the most used classification system, but using the embryological–clinical classification to analyze descriptions of female genital anomalies published in case reports, we have been able to classify 89.9% of cases, as well as 86.5% of cases defined as unclassifiable in those studies. Therefore, the embryological–clinical classification is highly applicable. Similarly, we have shown the completeness and higher accuracy of this classification, which makes it possible to specify and subclassify more cases than the other systems. As such, the embryological–clinical classification is considered more suitable.

## Figures and Tables

**Table 1:** Classification of cases as reported in the articles.

Total Number of Cases	824 (100%)		
**Classified using**	159 (19.3%)	AFS classification	101 (63.5%)
		Embryological–clinical classification	20 (12.6%)
		ESHRE/ESGE classification	16 (10.1%)
		More than one classification system	22 (13.8%)
**Unclassifiable with**	52 (6.3%)	AFS classification	32 (61.5%)
		ESHRE/ESGE classification	1 (1.9%)
		Any classification system	5 (9.6%)
		An unknown classification system	4 (7.7%)
**Not classified**	613 (74.4%)		

AFS: American Fertility Society. ESHRE/ESGE: European Society of Human Reproduction and Embryology/European Society for Gynaecological Endoscopy.

**Table 2:** Classification of the cases using the embryological–clinical classification [6].

Total Number of Cases	824 (100%)	
**Classified**	741 (89.9%)	
**Inconclusive**	63 (7.6%)	Lack of anatomical data in the text to distinguish between two types of malformation
**Not classified**	20 (2.4%)	4—Genital malformation not demonstrated
		16—Insufficient description of the malformation in the text

**Table 3:** Classification of the cases reported as unclassified by their authors using the embryological–clinical classification [6].

Reference	Patients	Uterus	Cervix	Vagina	Renal Agenesis	Author’s Definition	Embryological–Clinical Classification [6]	Comments Our Description
**Caliskan et al. (2008) [106]**	1	Septate	Septate	Longitudinal vaginal septum	No	The classification of this disorder is a subject of controversy.	3.A.5-1	Septate uterus.
**Celik et al. (2012) [105]**	1	Septate	Double	Longitudinal vaginal septum	Not investigated	A Müllerian anomaly without classification.	3.A.5-1	Septate uterus.
**Di Spiezio et al. (2007) [104]**	1	Normal	Normal	Partial longitudinal vaginal septum	Not investigated	Longitudinal vaginal septum.	3.B.2	Müllerian tubercle anomaly, resorption defect.
**Diehl et al. (2009) [10]**	1	Right unicornuate uterus with rudimentary, cavitated left horn that is non-communicating.	Single	Single	Left	Unusual Müllerian Anomaly.	2.5	Unicornuate uterus with contralateral unattached but cavitated rudimentary horn and ipsilateral renal agenesis. Ruptured left hematosalpinx.
**Duhan et al. (2016) [103]**	1	Left unicornuate uterus with cavitated right horn that is non-communicating.	Single (communicated with left cavity)	Normal	Not investigated	Unclassified Müllerian variant.	3.A.4	Bicornis-unicollis uterus with a non-communicating cavitated uterine horn. Patient with 4 laparotomies, 2 cesarean sections. It is possible that it is a septate/subseptate uterus (3.A.5) and the septum and uterine wall were sutured during second cesarean section, closing the right hemicavity.
**Duffy et al. (2004) [102]**	1	Septate	Double	Longitudinal vaginal septum	Not investigated	Rare Müllerian duct malformation.	3.A.5-1	Septate uterus.
**Dunn et al. (2004) [101]**	1	Normal	Septate (only left cervix communicates with the uterine cavity)	Longitudinal vaginal septum	No	Rare Müllerian anomaly.	3.B.2	Müllerian tubercle anomaly, cervico-vaginal fusion and resorption defects.
**El Saman et al. (2011) [100]**	1	Bicornuate, with a normal left hemi-cavity, and a non-communicating, cavitated right horn.	Single (communicated with left cavity)	Normal	No	Unclassified new anomaly.	3.A.4-2	Bicornis-unicollis uterus with a non-communicating cavitated uterine horn.
**Engmann et al. (2004) [99]**	1	Unicornuate	Single	Normal	Not investigated	It has not been previously included in the classification of AFS. They propose inclusion of this anomaly as a subcategory under Type II.	3.A.2	Unicornuate uterus with atretic non-cavitated rudimentary horn, or segmentary atresia.
**Fedele et al. (2012) [98]**	1	Septate	Septate	Septate + imperforated hymen	No	The combination of a uterovaginal septum with an imperforated hymen does not seem to fit into the existing classification systems.	6 (3.A.5-1 + 5)	Malformative combination: Septate uterus + anomalies of the urogenital sinus.
**Frontino et al. (2009) [97]**	1	Unicornuate uterus with an occult cavitated rudimentary horn.	Normal	Normal	No	Unusual presentations do not fit into this system (AFS).	3.A.2-1	Unicornuate uterus with cavitated non-communicated right uterine horn.
	1	Left unicornuate uterus and right uterine nodule.	Normal	Normal	Not investigated	Unusual presentations do not fit into this system (AFS).	3.A.2-1	Unicornuate uterus with cavitated non-communicated right uterine horn.
**Garofalo et al. (2017) [96]**	1	Normal uterus with accessory and cavitated uterine mass.	Normal	Normal	Not investigated	ACUM. Unusual presentations still do not fit into this system (AFS, ESHRE).	4	Accessory and cavitated uterine masses with normal uterus.
**Gholoum et al. (2006) [95]**	10	Didelphys	Double	Blind hemivagina (7 right, 3 left)	7 right, 3 left	HWW syndrome.	2.1	Uterine duplicity with hematocolpos in blind hemivagina and ipsilateral renal agenesis.
	1	Didelphys	Double	Blind hemivagina + partially obstructing contralateral vaginal septum	Right	HWW syndrome + other pathologies.	6 (2.1 + 3.B.2)	Malformative combinations: Uterine duplicity with a blind hemivagina and ipsilateral renal agenesis + contralateral incomplete transverse vaginal septum.
	1	Didelphys (without communicating uteri)	Single	Single	Right	HWW syndrome + cervical atresia.	2.5	Uterine duplicity with complete unilateral cervico-vaginal atresia, hematometra and ipsilateral renal agenesis.
**Goluda et al. (2006) [94]**	1	Bicornuate rudimentary uterine horns with functioning endometrium.	Absent	Absent	No	This case cannot be assigned to any group of the AFS classification.	3.C	Rokitansky syndrome with rudimentary uterine horns and endometriosis. Hereditary renal cystic syndrome.
**Guo et al. (2011) [93]**	1	Septate	Double	Longitudinal vaginal septum	Not investigated	This unique type of müllerian anomaly does not fall into the AFS classification.	3.A.5-1	Septate uterus.
**Gupta et al. (2007) [92]**	1	Asymmetric septate uterus with non-communicating right hemicavity.	Single	Normal	No	A unique congenital Müllerian anomaly: Robert’s uterus.	3.A.2-1	Unicornuate uterus with atretic cavitated rudimentary horn.
**Hundley et al. (2001) [91]**	1	Bicornuate	Double	Double vagina with partial longitudinal vaginal septum	No	This unusual müllerian anomaly does not fit in the commonly used classification system suggested by Buttram and Gibbons.	3.A.4-1	Bicornis-bicollis uterus with vaginal longitudinal septum.
**Iglesias-Lopes et al. (2014) [87]**	1	Bicornuate	Single	Uterovesical fistula + anomaly of the urogenital sinus (repaired at childhood)	Right	Complex Müllerian abnormality that cannot be assigned to any group of this classification (AFS).	6 (2.4 + 5)	Malformative combination: Uterine duplicity with complete unilateral cervico-vaginal atresia with communicating uteri and ipsilateral renal agenesis + anomaly of the urogenital sinus (imperforated anus with anorectoplasty in childhood).
**Kisu et al. (2014) [90]**	1	Normal uterine body, separated from cervix.	Normal	Normal	No	This case of “disconnected uterus” did not correspond to the conventional classification (ESHRE).	3.B.2	Anomalies of the connection of the Müllerian ducts with the Müllerian tubercle. Isthmic segmentary atresia.
**Kumar et al. (2008) [89]**	1	Double uterus with cavitated horns.	Absent	Agenesis + vesicouterine fistula.	No	This case report cannot be assigned to any group of the AFS classification.	6 (3.C + 5)	Malformative combination: Rokitansky syndrome with cavitated horns + left horn with vesical pseudofistula.
**Lima et al. (2013) [88]**	1	Bicornuate	Single	Single	Left	Hybrid Müllerian Duct Anomaly. They propose that the AFS classification of these anomalies should be revised.	2.5	Uterine duplicity with complete unilateral cervico-vaginal atresia without communicating uteri (left hematometra and hematosalpinx) and ipsilateral renal agenesis.
**Marques et al. (2013) [86]**	1	Rudimentary didelphic uterus with fibroids.	Absent	Normal	No	Unclassified uterine anomaly.	3.A.1	Agenesis of both Müllerian ducts. Seven cm vagina. Normal right kidney. Duplex left kidney.
**Medema et al. (2008) [85]**	1	Tricavitated	Single	Normal	Not investigated	This tricavitated anomaly of the uterus cannot be clearly explained, according to the classification for uterine malformations by the AFS.	3.A.7	Tricavitated uterus.
**Pavone et al. (2006) [84]**	1	Septate	Double	Longitudinal vaginal septum	No	A Müllerian anomaly without a classification	3.A.5-1	Septate uterus.
**Sadik et al. (2002) [83]**	1	Tricavitated and rudimentary uterus	Hypoplastic	Normal	No	Unknown anomaly of the uterus	3.A.7	Tricavitated uterus. Possible DES syndrome.
**Samad et al. (2000) [82]**	1	Bicornuate	Undetermined	Common chamber. Cloaca	Not investigated	Cloacal anomalies. Currently there is no universally accepted classification system.	6 (3.A.4 + 5)	Malformative combination: Anomalies of the urogenital sinus + bicornuate uterus.
	1	Normal	Undetermined	Common chamber. Cloaca	Not investigated	Cloacal anomalies. Currently there is no universally accepted classification system.	5	Anomaly of the urogenital sinus.
**Shirota et al. (2009) [81]**	1	Normal	Double	Septate	No	A Müllerian anomaly without any present classification.	3.B.2	Müllerian tubercle anomaly, cervico-vaginal fusion and resorption defects.
**Tanaka et al. (2013) [80]**	1	Arcuate	Normal	Normal	No	Complex Müllerian malformation without any present classification.	3.A.6	Arcuate uterus. Right dermoid cyst. Absence of the left ovary and fallopian tube. It may be due to ischemia and atrophy from previous adnexal torsion.
**Varras et al. (2007) [79]**	1	Normal	Double	Septate	No	Unusual Müllerian anomaly.	3.B.2	Müllerian tubercle anomaly, cervico-vaginal fusion and resorption defects. Fibroid uterus.
**Wenz et al. (2020) [78]**	1	Didelphys + cloacal exstrophy	Double	Left blind hemivagina	Horseshoe kidney	HWW syndrome. Multiple congenital anomalies.	6 (2.1 + 5)	Malformative combination: Uterine duplicity with a blind hemivagina and renal anomaly + anomaly of the urogenital sinus. Patient with multiple surgeries in childhood, it is difficult to know which anomalies are congenital and which are secondary.
**Wright et al. (2011) [77]**	1	Cavitated and rudimentary horns, not connected to the cervix	Normal	Normal	Pancake pelvic kidney	Unusual reproductive tract anomaly which is challenging to explain from an embryologic standpoint.	3.A.1	Hypoplasia of both Müllerian ducts. It seems to have an associated mesonephric anomaly due to pancake pelvic kidney.
**Yang et al. (2015) [76]**	1	Normal uterine body, isthmic agenesis	Normal	Normal	No	This case is exceedingly rare and hard to classify according to the AFS classification.	3.B.2	Anomalies of the connection of the Müllerian ducts with the Müllerian tubercle. Isthmic segmentary atresia.

AFS: American Fertility Society. ACUM: Accessory and cavitated uterine masses. ESHRE: European Society of Human Reproduction and Embryology. HWW: Herlyn-Werner-Wünderlich Syndrome.

**Table 4:** Dispersion matrix.

		AFS Classification								
		I	Ia	Ia + Ib	Ib	Ie	II	IIb	III	IV
**Embryological–Clinical** **Classification**	1.2						1			
	2.1								22	1
	2.1 + 5								1	
	2.3								5	
	2.5						2	3	1	
	2.5 + 5							1		
	3.A.2						4	17		
	3.A.3								1	
	3.A.4							5		1
	3.B.1			1	9			1		
	3.B.1 + 3.A.4				1					
	3.B.2	1	1		4		2			

AFS: American Fertility Society.

## Data Availability

The raw data supporting the conclusions of this article will be made available by the authors on request.

## Supplementary Materials

The following supporting information can be downloaded at: https://www.mdpi.com/article/10.3390/jcm13102988/s1, Supplementary Materials: Embryological-clinical classification.
