# Supplementary material for: Applicability and Suitability of the Embryological–Clinical Classification of Female Genital Malformations: A Systematic Review
Source: J Clin Med. 2024 May 19;13(10):2988. doi: 10.3390/jcm13102988 (PMC11121905; doi:10.3390/jcm13102988)
Supplement: Supplementary file 1 [file jcm-13-02988-s001.zip › jcm-2944913-supplementary.pdf]

Appendix A - Embryological-clinical classification

Embryological-clinical classification for female genito-urinary malformations (2011) [6]

1. Unilateral genito-urinary agenesis or hypoplasia

1.1 With contralateral müllerian agenesis
1.2 Without contralateral agenesis

2. Uterine duplicity with a blind hemivagina (or atresia) and ipsilateral RA, showing

2.1 Large hematocolpos, blind hemivagina. Didelphys or bicornuate uterus with blind hemivagina and ipsilateral RA
2.2 Like Gartner's pseudocyst. Bicornuate communicating uterus, atretic blind hemivagina and ipsilateral RA. Herlyn-Werner syndrome
2.3 Partial reabsorption of the vaginal septum. Didelphys or bicornis-bicollis uterus with a short vaginal septum or buttonhole, and URA
2.4 Complete unilateral vaginal or cervico-vaginal atresia with communicating uteri. Bicornis-unicollis uterus with an anomalous horn and ipsilateral RA
2.5 Idem, without communicating uteri. Unicornuate uterus with contralateral unattached but cavitated rudimentary horn and URA

3. Isolated or common uterine or utero-vaginal anomalies, affecting

A. Paramesonephric or müllerian ducts:
A.1. Agenesis or hypoplasias. Müllerian agenesis
A.2. Unicornuate uterus with atretic cavitated or non-cavitated rudimentary horn, or bicornuate with cavitated noncommunicated uterine horn, or segmentary atresia, or 'unilateral Rokitansky syndrome'
A.3. Didelphys uterus
A.4. Bicornuate uterus. Eventually, with a non-communicating cavitated uterine horn
A.5. Septate uterus
A.6. Arcuate uterus
A.7. Anomalies related to DES syndrome. DES syndrome. Hypoplastic and T-shaped uterus. Tricavitated uterus

B. Müllerian tubercle
B.1. Complete vaginal or cervico-vaginal agenesis or atresia
B.2. Segmentary atresias. Complete or incomplete transverse vaginal septum

C. Both Müllerian tubercle and ducts:
Complete utero-vaginal agenesis. Rokitansky or MRKH syndrome

4. Accessory uterine masses and other gubernaculum dysfunctions.

Accessory and cavitated uterine masses (ACUM) with normal uterus. Didelphys uterus without RA?

5. Anomalies of the urogenital sinus.

Imperforated hymen. Persistent urogenital sinus. Congenital vesico-vaginal fistula, cloacal anomalies, and other external gastrointestinal or urinary anomalies

6. Malformative combinations

URA: unilateral renal agenesis.
RA: renal agenesis
MRKH: Mayer–Rokitansky –Kuster–Hauser.
DES: diethylstilbestrol.
